# Sinkhole susceptibility mapping in Marion County, Florida: Evaluation and comparison between analytical hierarchy process and logistic regression based approaches

**DOI:** 10.1038/s41598-019-43705-6

**Published:** 2019-05-09

**Authors:** Praveen Subedi, Kabiraj Subedi, Bina Thapa, Pradeep Subedi

**Affiliations:** 1 School of Forest Resources and Conservation, University of Florida, Gainesville, Florida, USA; 2 Department of Geography, Prithivi Narayan Campus, Tribhuvan University, Pokhara, Nepal; 3 Department of Natural Resource Ecology and Management, Iowa State University, Ames, Iowa USA; 4 Rutgers Discovery Informatics Institute, Rutgers University, Piscataway, New Jersey USA

**Keywords:** Natural hazards, Solid Earth sciences

## Abstract

Sinkholes are the major cause of concern in Florida for their direct role on aquifer vulnerability and potential loss of lives and property. Mapping sinkhole susceptibility is critical to mitigating these consequences by adopting strategic changes to land use practices. We compared the analytical hierarchy process (AHP) based and logistic regression (LR) based approaches to map the areas prone to sinkhole activity in Marion County, Florida by using long-term sinkhole incident report dataset. For this study, the LR based model was more accurate with an area under the receiver operating characteristic (ROC) curve of 0.8 compared to 0.73 with the AHP based model. Both models performed better when an independent future sinkhole dataset was used for validation. The LR based approach showed a low presence of sinkholes in the very low susceptibility class and low absence of sinkholes in the very high susceptibility class. However, the AHP based model detected sinkhole presence by allocating more area to the high and very high susceptibility classes. For instance, areas susceptible to very high and high sinkhole incidents covered almost 43.4% of the total area under the AHP based approach, whereas the LR based approach allocated 20.7% of the total area to high and very high susceptibility classes. Of the predisposing factors studied, the LR method revealed that closeness to topographic depression was the most important factor for sinkhole susceptibility. Both models classified Ocala city, a populous city of the study area, as being very vulnerable to sinkhole hazard. Using a common test case scenario, this study discusses the applicability and potential limitations of these sinkhole susceptibility mapping approaches in central Florida.

## Introduction

In the United States, soluble rocks underlie almost 18% of the total area and have the potential to develop karstic features^1^. Dissolution of these underlying rocks often results in the collapse of overlying structures forming sinkholes. Between 2000 and 2014, a conservative estimate of sinkhole damage costs for the United States was at least 300 million dollars per year with the actual cost being much higher^2^. In Florida alone, sinkhole-related claims increased by 3-folds totaling a valuation of about 1.4 billion dollars between 2006 and 2010^3^. Like economic effects, environmental effects of sinkholes are also critical in Florida where over 10 million people depend on groundwater^4^. Sinkholes form flow channels that potentially direct flow of surface water into aquifers without adequate filtration and contaminate water^5,6^. Management of these economic and environmental hazards requires a detailed study of sinkhole formation, predisposing factors for sinkhole development, and mapping of areas vulnerable to sinkholes.

Previous studies on sinkholes show that precipitation, soil types, underlying geology, water channels, faults and folds, slope, karst topography, fluctuation of the water table, and thickness of the overburden affect their formation^7–12^. Furthermore, anthropogenic factors like ground-water pumping and mining accelerate sinkhole development^10^. Identifying complex interactions between these factors forms a basis for sinkhole susceptibility mapping^8,13–15^. More importantly, factors like data availability, spatial extent, and choice of modeling approaches remain critical in improving the reliability of sinkhole susceptibility mapping. A majority of sinkhole susceptibility mapping approaches use qualitative and/or quantitative techniques. Comparison of these techniques in a common setting provides important inferences on the benefits and pitfalls of these modeling approaches.

Common quantitative modeling approaches to sinkhole susceptibility mapping include deterministic, nearest neighbor or density distribution, and probabilistic methods^8,16–20^. Deterministic methods take into account factors that contribute to the stability of an area with respect to sinkhole formation^21,22^. This approach may become less practicable in extensive areas as costs constrain collections of many parameters that affect geometric stability of land surface. The nearest neighbor or density distribution methods take into account the spatial distribution of existing sinkholes and assigns higher importance to neighboring sinkholes in the formation of new sinkholes^14,19^. This method may not be practical in situations where underlying geological feature constraining sinkhole formation is highly directional (e.g., linear)^23^. Probabilistic methods use a statistical relationship between predisposing factors and the existing events of sinkholes to map sinkhole susceptible zones^8,9,24^. Previous studies of susceptibility mapping of natural hazards have commonly used bivariate and multivariate statistical analysis^25–28^. Recently, logistic regression (LR) approach has been popular among susceptibility modelers mainly because it works well with both categorical and continuous variables^23,29–31^.

Qualitative or heuristic approaches, on the other hand, use a subjective scoring system based on the degree of contribution of a factor to form sinkholes^17,20^. While approaches rely on expert judgment unlike quantitative methods, the relative simplicity and flexibility make them suitable for regional assessments. However, hybrid approaches (or semi-heuristic approaches) like weighted linear combination are also being used in susceptibility mapping^27,32^. In recent years, the analytical hierarchy process (AHP) has been widely used in susceptibility mapping as it allows for: (a) decomposition of component factors (e.g., predisposing factors) into several sub-classes or sub-criteria, (b) hierarchical arrangement of these factors, (c) pairwise comparison among and within predisposing factors, and (d) synthesis of comparisons to obtain susceptibility metrics^27,33–37^.

Despite complexities in susceptibility estimations, the combination of these qualitative and quantitative methods with Geographic Information System (GIS) significantly simplifies the mapping of sinkhole susceptible areas^38^. As a result, there has been a growing interest combining these approaches with GIS to map susceptibility of environmental hazards^7,8,25,28,29,39–41^. Using over four decades of sinkhole incidence data available for central Florida, we intend to produce the spatially explicit map of sinkhole susceptible areas. Further, we compare two widely used methods of sinkholes mapping- AHP based and LR based approaches. While these methods have been developed and implemented for a particular case and region, here, we attempt to review and quantitatively evaluate the efficiency of methods in the same case scenario. This study would provide the robustness of model calibration and provides useful information for further enhancements. Furthermore, we demonstrate the advantages and potential limitations of these mapping approaches in the context of central Florida and suggest areas for improvement. In addition, this study also investigates the importance of factors both natural (e.g., proximity to closed topographic depression in karst topography, surficial geology, soil permeability, proximity to the flow channels/drainage networks) and anthropogenic (e.g., active mines) in nature in mapping sinkhole susceptible zones.

## Materials and Methods

### Study area

**Figure 1:**
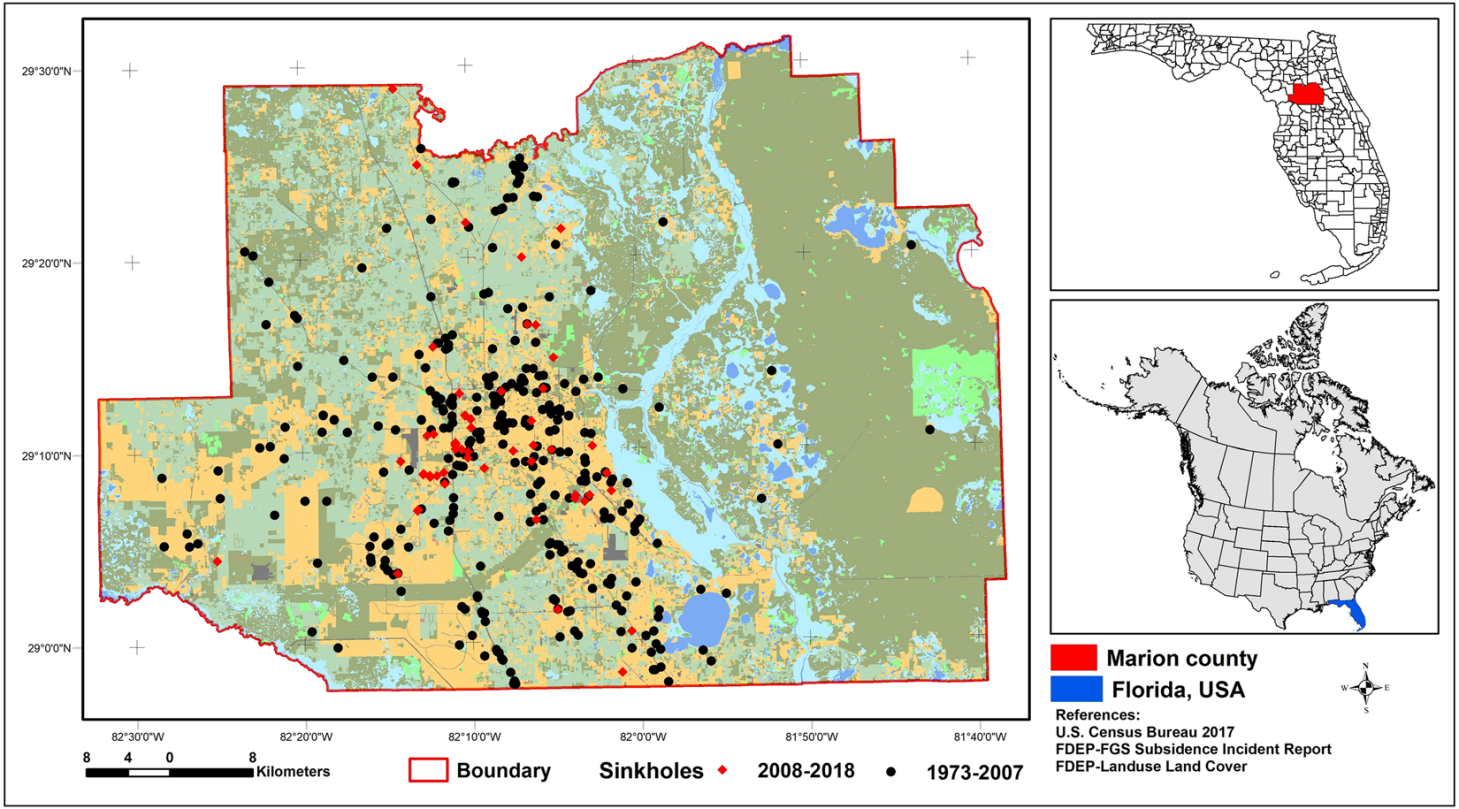
Location of the study area and sinkhole incidents in Marion County, Florida, USA. The basemap is a land use land cover map prepared using the publicly available land use land cover data for Florida. The land use land cover data is prepared and maintained by the Florida Department of Environmental Protection (2017) and is publicly available online at: http://publicfiles.dep.state.fl.us/otis/gis/data/STATEWIDE_LANDUSE.zip. We used ArcMap 10.3.1 software (Environmental Systems Research Institute Inc., Redlands, CA, 2015) for the preparation of this map.

Marion County is located in North-Central Florida (Fig. 1). Mean annual temperature and rainfall of the county are 21.8 °C and 1290 mm respectively. The county is densely populated with a density of 81 persons per sq. km^42^. The population growth rate of Marion County between 2000 and 2010 was higher than the statewide growth rate in that period^42^ and will continue to increase in the future. Florida Department of Community Affairs (2005)^43^ also assumes that almost 30% of the total population in Marion County is living under immediate threats of sinkhole formation. Likewise, 32,260 building structures in Marion County are also assumed to be under the risk of sinkhole formation^43^. In that context, identifying the potential areas that are susceptible to sinkhole formation is critical to formulate strategic land-use guidelines and hazard response mechanisms to avoid serious losses of structures and human lives.

### Hydrogeology of the study area

The underlying geology of the county consists predominantly of Ocala limestone (Eocene; 30% area). Ocala limestone comprises of pure limestones and occasional dolomites and is highly permeable component of the Florida aquifer system^44^. Cypresshead formation (Pliocene) covers about 26% of the study area and consists of unconsolidated to poorly consolidated sands. It is a permeable component of the surficial aquifer system. Coosawhatchie formation (Miocene; 17%) is a relatively less permeable component of the intermediate confining unit. It consists of unconsolidated clayey and phosphatic sands to moderately consolidated sandy clay^45^. Undifferentiated sediments (Pleistocene/Holocene) cover about 10% of the area and consist of silica-rich rocks, organics, and freshwater carbonates. Undifferentiated tertiary-quaternary sediments (Pliocene/Pleistocene; 3% of total area) consist of poorly consolidated sands, sandy clays or clays, and organic debris. Undifferentiated sediments are also the components of surficial aquifer system. Holocene sediments (Holocene; 6% of total area) consist of quartz sands, carbonate sands, and organics. Hawthorn formation undifferentiated (Miocene) covers about 3% of the study area and consists of highly weathered clayey sands to silty clays poor in phosphates. It is also an important component of the intermediate confining unit of the Florida aquifer system.

### Identification of data layers

Factors like karst topography^17,39^, surficial geology^16,24^, soil permeability^46^, proximity to the flow channels or drainage networks^8,17^, groundwater withdrawal^47^, depth to the water table^9,48^, mining activity^49^, the thickness of overburden^20^, recharge of aquifers^50^ etc., are thought to influence the formation of sinkholes. For this study, proximity to closed topographic depression (karst topography), surficial geology, soil permeability, proximity to the flow channels/drainage networks, and the proximity to the active mines were considered as the predisposing factors for sinkhole formation. Details on the data layers used and sources are provided in Table 1. Selection of these data layers was based on their relevance to sinkhole formation and their availability for the whole study area. Sinkhole inventory data for the study area was extracted from a publicly available subsidence incident report maintained by the Florida Department of Environmental Protection (FDEP) - Florida Geological Survey (FGS) available online at: http://publicfiles.dep.state.fl.us/otis/gis/data/FGS_SUBSIDENCE_INCIDENTS.zip.

**Table 1:** Data Layers used for sinkhole susceptibility zonation.

Data Layers	Source	Scale	Publication Date
**Surficial Geology**	Florida Department of Environmental Protection-Florida Geological Survey	1: 100,000	1998–2001/updated on 2017
**Closed Topographic depressions in Florida**	Florida Geological Survey	1: 24,000	2015
**Soil Permeability**	Florida Department of Environmental Protection- Florida Geological Survey (data from National Resource Conservation Service, USDA)	30 m × 30 m Raster Digital Data	2006
**Flowlines**	United States Geological Survey *(National Hydrographic Dataset)*	1: 24, 000	2017
**Active Mines**	Florida Geological Survey	N/A	2006
County Boundary	United States Census Bureau- TIGER/Linefiles	1: 100,000	1999
Sinkholes	Florida Department of Environmental Protection- Florida Geological Survey *Subsidence Incident report*	1: 24,000	2015/updated monthly
Statewise Land Use Land Cover	Florida Department of Environmental Protection	N/A	2017

Note: Bold data layers were used as predisposing factors in sinkhole susceptibility modeling.

### Analytical hierarchy process based approach to susceptibility mapping

#### Preparation of data layers and sub-criteria

**Figure 2:**
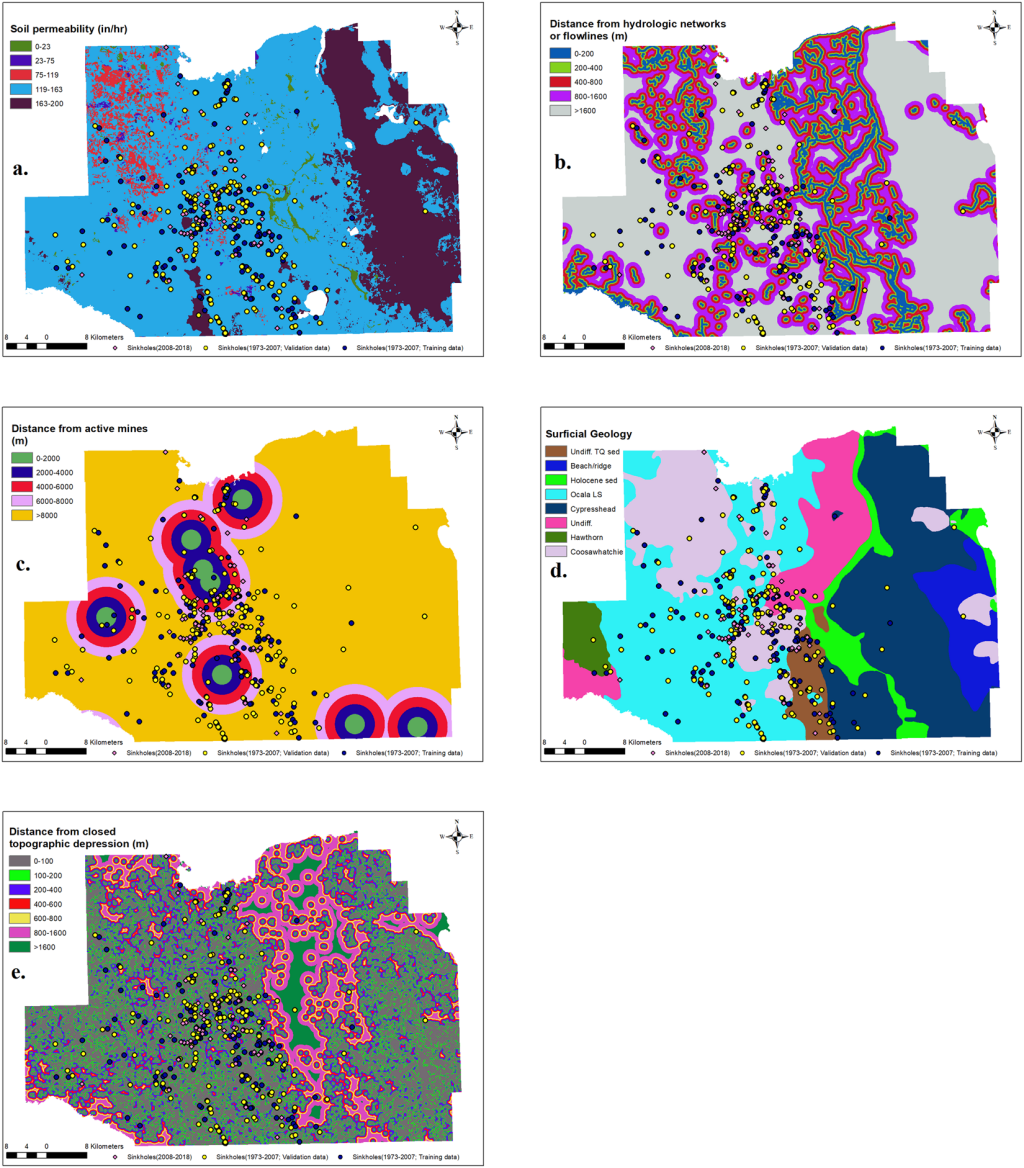
Data layers and subclasses: (**a**) soil permeability (in/hr), (**b**) Distance from hydrologic networks (m), (**c**). Distance from active mines (m), (**d**) Surficial geology, and (**e**) Distance from closed topographic depressions (m).

Soil permeability: Rawal (2016)^51^ showed a linear relationship between the time for soil surface collapse and soil permeability in a simulated sinkhole study. Soil permeability raster data was originally prepared from the soil survey geographic data (SSURGO) maintained by the USDA-Natural Resources Conservation Service. Briefly, polygons present in the soil permeability data shapefiles obtained from the USDA-NRCS were dissolved based on permeability value and converted into raster file to create soil permeability raster data by the Florida Geological Survey. We used this 30 m × 30 m soil permeability raster digital data, available from the Florida Geological Survey, as soil permeability data layer for the study area (Florida Geological Survey 2006^52^). Soil permeability raster map was reclassified manually into five classes; 0–23, 23–75, 75–119, 119–163, and 163–200 in/hr (Fig. 2a) in ArcGIS 10.3.1 software (Environmental Systems Research Institute, Inc., Redlands, CA, 2015), and the areas that had no soil permeability value were left as no value pixels in our analysis. The frequency ratio of sinkhole incidents in these classes suggested that with an increase in soil permeability, sinkhole incidents increased in general (Table 2). More importantly, most sinkholes occurred in soils with the permeability of 130 in/hr for this study area.

**Table 2:** Number, percentage, and frequency ratio distribution of sinkholes (1973–2007) (n = 337) in data layers and sub-criteria.

Sub-criteria in data layers	Total Area (Km^2^)	Percent total area (a)	No. of sinkholes	Percent total no. of sinkholes (b)	Frequency ratio (b/a)
***Soil permeability (in/hr)***					
0–23	47.66	1.12	0	0.00	0.000
23–75	23.58	0.55	1	0.30	0.539
75–119	176.50	4.14	10	2.97	0.717
119–163	3084.24	72.42	319	94.66	1.307
163–200	926.64	21.76	7	2.08	0.095
***Distance from flowlines (m)***					
0–200	395.94	9.19	12	3.56	0.387
200–400	349.57	8.12	24	7.12	0.877
400–800	645.91	15	63	18.69	1.246
800–1600	976.34	22.67	99	29.38	1.296
>1600	1938.52	45.02	139	41.25	0.916
***Distance from active mines (m)***					
0–2000	98.44	2.29	10	2.97	1.296
2000–4000	256.96	5.97	33	9.79	1.640
4000–6000	367.06	8.52	52	15.43	1.811
6000–8000	434.56	10.09	66	19.58	1.941
>8000	3149.26	73.13	176	52.23	0.714
***Surficial geology***					
Ocala LS	1290.45	29.97	145	43.03	1.436
Coosawhatchie FM	744.97	17.30	104	30.86	1.784
Undifferentiated TQ sediments	142.69	3.31	33	9.79	2.958
Undifferentiated	417.84	9.70	30	8.90	0.917
Cypresshead FM	1110.51	25.79	20	5.93	0.230
Hawthorn FM	108.07	2.51	4	1.19	0.472
Holocene sediments	242.19	5.62	1	0.30	0.053
Beach ridge & dune	249.55	5.80	0	0.00	0.000
***Distance from closed topographic depression (m)***					
0–100	1749.14	40.62	206	61.13	1.504
100–200	757.78	17.6	61	18.10	1.028
200–400	761.98	17.7	55	16.32	0.922
400–600	328.98	7.6	10	2.97	0.390
600–800	192.75	4.5	3	0.89	0.198
800–1600	373.27	8.7	1	0.30	0.034
>1600	142.38	3.3	1	0.30	0.090

Distance from flowlines: Previous studies have shown that irrigation networks and hydrologic flow channels are important predictors of cover-collapse sinkholes^23^. We used flowlines shapefile in the national hydrography dataset prepared at the scale of 1:24000 by the United States Geological Survey in 2012 available at: https://prd-tnm.s3.amazonaws.com/StagedProducts/Hydrography/NHD/State/HighResolution/Shape/NHD_H_Florida_State_Shape.zip. We created the distance from flowlines data layer with a 30 m × 30 m resolution from the flowline vector file by using the Euclidean distance function in the Spatial Analyst tool in ArcGIS 10.3.1 software (Environmental Systems Research Institute, Inc., Redlands, CA, 2015). For this study, we observed higher sinkhole occurrence near the hydrological flow networks. The cumulative distribution function of sinkholes showed a linear increase in cumulative probability up to 1600 m and after that, it started to increase at a decreasing rate. We manually reclassified the raster map of distance from flowlines into five classes; 0–200, 200–400, 400–800, 800–1600, >1600 m (Fig. 2b).

Distance from active mines: Mining activity is generally associated with the formation of sinkholes because of weak overburden, geological discontinuities, and dissolution of exposed rocks^11,49,53^. We used active mines shapefile available from Florida Geological Survey (2006). This data contained active mine point features for the US Department of Interior, Mine Safety and Health Administration Retrieval Data System as of June 2006. We used active mines shapefile to create the distance from active mine raster using Euclidean distance function available in the Spatial Analyst module in ArcGIS 10.3.1 software (Environmental Systems Research Institute, Inc., Redlands, CA, 2015). For this study site, the cumulative distribution function of sinkholes showed a linear increase up to 8000 m from active mines. We manually reclassified the distance from active mines layer into five sub-criteria; 0–2000, 2000–4000, 4000–6000, 6000–8000, >8000 m (Fig. 2c).

Surficial geology: Underlying geology has been one of the important factors considered in most sinkhole susceptibility studies^16,17,24,39^. Usually, sinkholes develop in regions where underlying bedrocks made of limestones, carbonate rocks, or salt beds are likely to dissolve. Stratigraphic geology vector data file (last updated in 2017) from the Florida Geological Survey (2001) available online at: http://publicfiles.dep.state.fl.us/OTIS/GIS/data/GEOLOGY_STRATIGRAPHY.zip was used to extract surficial geology polygons for the study area. We converted the surficial geology data layer to a raster with a pixel size of 30 m × 30 m based on the geologic formations in the Marion County. We used the Spatial Analyst tool available in ArcGIS 10.3.1 (Environmental Systems Research Institute, Inc., Redlands, CA, 2015) for raster conversions. Eight geological types present in the surficial geology data layer were chosen as sub-criteria; Ocala limestone (LS), Coosawhatchie formation (FM), Undifferentiated tertiary-quaternary (TQ) sediments, Undifferentiated, Cypresshead formation (FM), Hawthorn formation (FM), Holocene sediments, and Beach ridge & dune (Fig. 2d). The frequency ratio of sinkhole occurrence suggested that Ocala limestone, Coosawhatchie formation, and undifferentiated tertiary-quaternary sediments had higher sinkhole occurrence than other geological sub-criteria (Table 2).

Distance from topographic depressions: Closeness to topographic depression has been related to sinkhole occurrence in previous studies^54^. We obtained elevation and contours dataset shapefiles available at: http://publicfiles.dep.state.fl.us/otis/gis/data/LAND_SURFACE_ELEVATION_24.zip prepared by Florida Geological Survey (2015). These shapefiles were digitized from the original US Geological Survey of land elevations at 1:24,000 scale (1980). We used closed topographic depression features available in this dataset for this study. To create a distance from closed topographic depressions map with a pixel size of 30 m × 30 m, Euclidean distance function in the Spatial Analyst in ArcGIS 10.3.1 (Environmental Systems Research Institute, Inc., Redlands, CA, 2015) was used. The cumulative distribution functions suggested a sharp initial increase in cumulative probability within 600 m of topographic depression and after that cumulative probability was increasing at a much slower rate. We manually reclassified the distance from topographic depressions layer into seven classes as, 0–100, 100–200, 200–400, 400–600, 600–800, 800–1600, and >1600 m (Fig. 2e). The frequency ratio of sinkhole occurrence based on this reclassification suggested that higher sinkholes occurred closer to topographic depressions (Table 2).

#### Pairwise comparison, decision matrix, and relative weights

Based on the scale of comparison prepared by Saaty (1980)^55^, we prepared pair-wise comparison matrix by relatively ranking the prevalence of one data layer or sub-criteria over the other in contributing sinkhole formation using subjective judgment (Table 3). The eigen vector associated with the principle eigen value of the pair-wise comparison matrix was used as weights^56^. We normalized the weights of eigen vectors prior to assignment to the data layers and sub-criteria. To evaluate the logical consistency of the pair-wise comparison between the data layers and sub-criteria, we used the consistency ratio defined as:

$$\frac{{\rm{C}}{\rm{.I}}{\rm{.}}}{{\rm{Random}}\,{\rm{C}}{\rm{.I}}{\rm{.}}},$$

where C.I. = Consistency Index = $\frac{{\lambda }_{{\rm{m}}{\rm{a}}{\rm{x}}}-{\rm{n}}}{{\rm{n}}-1}$, and λ_max_ is the principle eigen value of the pairwise comparison matrix, and n is the order of the matrix, and

**Table 3:** Scale of comparison^55^ used for pairwise comparison.

Score	Condition
1	Equal importance
3	Moderate prevalence of one over another
5	Strong or essential prevalence
7	Very strong or demonstrated prevalence
9	Extremely high prevalence
2, 4, 6, 8	Intermediate values
Reciprocal scores (1/2, 1/3..)	Inverse comparisons

Random C.I. = Consistency indices generated randomly for matrices with different orders^55^.

When the consistency ratio of the matrix exceeded 0.1, the matrix was not considered logical and reevaluation of the decision matrix was made. Subjective scoring of the relative prevalence of one sub-criterion over another and among data layers was based on the literature review and expert information. The relative weights of the sub-criteria and data layers are provided in Table 4.

**Table 4:** Pairwise comparison matrices and the obtained weights of sub-criteria and data layers.

Sub-criteria	Pairwise Comparison Matrices								Eigenvector associated with λ_max_	Normalized Weights
	1	2	3	4	5	6	7	8		
***Distance from closed topographic depressions (m)***										
0–100	1	3	5	6	7	8	9		1.000	0.428
100–200	1/3	1	2	3	5	7	8		0.496	0.213
200–400	1/5	½	1	3	5	7	7		0.388	0.166
400–600	1/6	1/3	1/3	1	2	5	5		0.203	0.087
600–800	1/7	1/5	1/5	½	1	2	4		0.118	0.051
800–1600	1/8	1/7	1/7	1/5	½	1	3		0.077	0.034
>1600	1/9	1/8	1/7	1/5	¼	1/3	1		0.050	0.021
	λ_max_ = 7.534			C.I. = 0.089			C.R. = 0.067			
***Surficial Geology***										
Ocala LS	1	2	3	5	7	7	8	9	1.000	0.338
Coosawhatchie FM	½	1	2	3	5	6	7	9	0.795	0.269
Undifferentiated TQ sediments	1/3	½	1	2	4	5	5	7	0.432	0.146
Undifferentiated	1/5	1/3	½	1	3	4	5	5	0.298	0.100
Cypresshead FM	1/7	1/5	¼	1/3	1	3	3	5	0.181	0.061
Hawthorn FM	1/7	1/6	1/5	¼	1/3	1	2	3	0.110	0.037
Holocene sediments	1/8	1/7	1/5	1/5	1/3	½	1	3	0.086	0.029
Beach ridge & dune	1/9	1/9	1/7	1/5	1/5	1/3	1/3	1	0.056	0.019
	λ_max_ = 8.598			C.I. = 0.089			C.R. = 0.061			
***Soil Permeability (in/hr)***										
0–23	1	½	1/3	1/7	1/7				0.144	0.046
23–75	2	1	½	1/5	1/5				0.235	0.075
75–119	3	2	1	1/3	1/3				0.411	0.132
119–163	7	5	3	1	2				1.327	0.426
163–200	7	5	3	½	1				1.000	0.321
	λ_max_ = 5.080			C.I. = 0.020			C.R. = 0.018			
***Distance from Flowlines (m)***										
0–200	1	2	3	4	4				1.000	0.410
200–400	½	1	2	3	3				0.621	0.254
400–800	1/3	½	1	2	3				0.401	0.165
800–1600	¼	1/3	½	1	2				0.244	0.100
>1600	¼	1/3	1/3	½	1				0.173	0.071
	λ_max_ = 5.114			C.I. = 0.028			C.R. = 0.025			
***Distance from active mines (m)***										
0–2000	1	1/3	¼	1/7	1/7				0.135	0.042
2000–4000	3	1	½	1/3	1/3				0.339	0.106
4000–6000	4	2	1	½	1/3				0.522	0.163
6000–8000	7	3	2	1	2				1.211	0.377
>8000	7	3	3	½	1				1.000	0.312
	λ_max_ = 5.136			C.I. = 0.034			C.R. = 0.030			
***Data layers***										
Closed topographic depressions	1	½	3	6	7				1.000	0.322
Surficial geology	2	1	3	6	7				1.324	0.426
Soil permeability	1/3	1/3	1	3	4				0.457	0.147
Flowlines	1/6	1/6	1/3	1	2				0.195	0.063
Active mines	1/7	1/7	¼	½	1				0.132	0.042
	λ_max_ = 5.126			C.I. = 0.031			C.R. = 0.028			

Where, λ_max_ is the principal eigen value; C.I. is the consistency index; and C.R. is the consistency ratio.

#### Sinkhole susceptibility mapping

**Figure 3:**
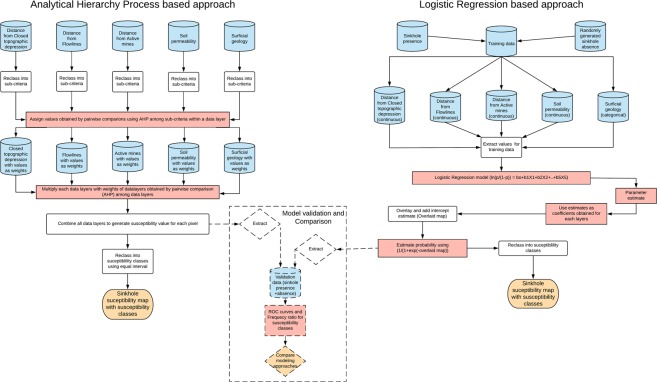
Workflow of susceptibility mapping using the AHP and LR based approaches and their evaluation.

We used weighted linear combination approach^57^ to map sinkhole susceptibility (S) = $\sum _{{\rm{i}}=1}^{{\rm{n}}}\,{{\rm{X}}}_{{\rm{i}}}\times {{\rm{W}}}_{{\rm{i}}}$ where, X_i_ is the relative weight of the sub-criteria of the i^th^ layer; and W_i_ is the relative weight of the i^th^ layer. Several approaches (quantile, standard deviation, natural breaks, or equal interval) to classify the pixel susceptibility values into susceptibility classes exist in the literature^17,27,32,34,58,59^. We used equal interval approach^27^ to classify the resulting map into five classes representing very low, low, medium, high and very high susceptibility to the formation of sinkholes. All spatial analyses were done using the Spatial Analyst tool in ArcGIS 10.3.1 software (Environmental Systems Research Institute, Inc., Redlands, CA, 2015). Flow chart of the AHP method is provided in Fig. 3.

### Logistic regression (LR) based approach to susceptibility mapping

A total of 398 subsidence events between November 1973 and June 2018 were identified as potential sinkholes for this study. Of which, a total of 61 sinkholes occurring between January 2008 and June 2018 were retained independently. Sinkhole incident data (n = 337) from 1973 to 2007 were divided randomly into two groups (almost a 50% split) to include in the training data (168) and validation data (169). We generated sinkhole absence data by randomly locating 700 data points within the study area (avoiding pixels with sinkhole incidence), of which 300 each were added to the training (n = 468) and validation (n = 469) data. Remaining 100 absence data points were added to the independent dataset (n = 161). We verified sinkhole absence data points post selection via field visit for easily accessible sites (Aug 2016–Mar 2017) and for inaccessible sites via use of digital ortho-rectified aerial imageries from 2008 (0.3 m × 0.3 m resolution; Florida Department of Revenue, 2008), multi temporal high resolution satellite images (1999–2018) from Google Earth Pro (0.15 m × 0.15 m resolution; Google Inc., 2018), and 1.5 m resolution digital elevation model data available for the study area (prepared by the Marion County IT/GIS team- Marion County using LiDAR data collected from 2003 to 2004) to make sure they did not represent potential sinkholes or sudden depressions. Sinkhole (training and validation) shapefiles were created from these data points for the Marion County.

Sinkhole presence and absence training data were used to construct a logistic regression model to map sinkhole susceptibility in our study. While it is a common practice in literature to use a balanced presence and absence data in logistic regression modeling of rare events^60^, numerous logistic regression based natural hazard susceptibility mapping studies have used unbalanced data^24,61–64^. Our preliminary analysis of the model comparison between under-sampling of the absence data to balance the dataset negatively influenced the model performance compared to the LR model generated using full dataset (unbalanced). We simulated under-sampled absence data for 10 times and generated a model average from the ten balanced LR models. We compared this model average to the LR model generated using full unbalanced dataset. This comparison showed that balancing the data would result in a decrease in model R^2^ (0.25 vs 0.32) value, an increase in root mean square error (RMSE: 0.44 vs 0.42), an increase in misclassification rate (0.31 vs 0.28), and a significant reduction in area under the receiver operating characteristic curve (AUC) (0.77 vs 0.78; pValue = 0.034, χ^2^-test at 95% confidence level) compared to using full but unbalanced dataset. Assigning higher weights to the presence data to balance the dataset did also reduce R^2^ value (0.25 vs 0.32), increase RMSE (0.44 vs 0.43), increase misclassification rate (0.31 vs 0.28), and reduce AUC (0.77 vs 0.78, pValue = 0.025, χ^2^-test at 95% confidence level) significantly compared to unbalanced dataset. The ROC curves for these analyses are provided in the supplementary information as Supplementary Fig. S1. Balancing the data by under-sampling may not always be reliable as it may result in loss of power of analysis^65^. For these aforementioned reasons, we used unbalanced dataset (presence:absence ratio = 1:1.78) for logistic regression in this study^63^.

A binary logistic regression method was applied for this study^31^. The model used for our LR based approach is:

$${\rm{Logit}}({\rm{p}})=\,\mathrm{ln}\,(\frac{{\rm{p}}}{{\rm{1}}-{\rm{p}}})={{\rm{b}}}_{0}+{{\rm{b}}}_{1}{{\rm{X}}}_{1}+{{\rm{b}}}_{2}{{\rm{X}}}_{2}+\ldots .+{{\rm{b}}}_{{\rm{n}}}{{\rm{X}}}_{{\rm{n}}},$$

where p is the probability of occurrence of a dependent variable, b_0_ is the intercept, b_1_, …, b_n_ are the coefficients of independent variables (X_1_, …, X_n_).

The probability of sinkhole occurrence (p) was estimated as:

$$1/(1+{\exp }^{-({{\rm{b}}}_{0}+{{\rm{b}}}_{1}{{\rm{X}}}_{1}+{\rm{\ldots }}+{{\rm{b}}}_{{\rm{n}}}{{\rm{X}}}_{{\rm{n}}})})$$

The presence and absence of sinkholes (binary data) was the dependent variable. Independent variables were the data layers which were either continuous (distance to flowlines, distance to active mines, distance to closed topographic depressions, and soil permeability) or categorical (Surficial geology). Multi-collinearity among independent were assessed by using a variable inflation factor (VIF) threshold of 10^66^. Pixel values (for the continuous variables) or attributes (for the categorical variables) were extracted for each presence and absence data points in ArcGIS 10.3.1 software (Environmental Systems Research Institute, Inc., Redlands, CA, 2015). The extracted values were then exported and LR modeling performed in JMP Pro14 software (SAS Institute Inc., Cary, NC, 2018).

The regression coefficient estimates obtained for each data layer using the training data were used to create a logistic regression model of sinkhole susceptibility for our study^24^. Flow chart of the methodology is provided in Fig. 3. The probability estimates for the study area was divided into five equal interval susceptibility classes (Very low, Low, Medium, High, and Very high) to prepare sinkhole susceptibility map.

### Evaluation of the AHP based and LR based models

We compared the distribution of susceptibility values generated by the models to the validation data of sinkhole presence and absence. We prepared the ROC curves for all the models using validation data and compared the area under the ROC curves to summarize the performance of the binary classifier (presence/absence of sinkholes)^24,28,36^. Model with the higher area under the ROC curve was considered a better performing model. We also performed a hypothesis test to compare if the AUCs differed significantly between AHP and LR based models at a 95% confidence level.

To evaluate the temporal suitability of the models^39,67^, we divided the validation data into three time periods based on the year of occurrence of sinkhole event in the study area (1974–1986; 1987–1998; and 1999–2007) and combined each of them with proportional sinkhole absence data (33%) in the validation dataset. Using this grouped validation data, area under the ROC curves were estimated for both models for three time periods. Likewise, to evaluate the suitability of the susceptibility mapping models prepared from past sinkhole incidents to predict future sinkhole susceptibility, we combined sinkhole incidents (n = 61) from 2008 to 2018 to random sinkhole absence data (n = 100) and calculated area under the ROC curves for both models. In addition, the frequency ratio of sinkhole presence or absence, defined as:

${\rm{F}}{\rm{r}}{\rm{e}}{\rm{q}}{\rm{u}}{\rm{e}}{\rm{n}}{\rm{c}}{\rm{y}}\,{\rm{R}}{\rm{a}}{\rm{t}}{\rm{i}}{\rm{o}}\,({\rm{p}}{\rm{r}}{\rm{e}}{\rm{s}}{\rm{e}}{\rm{n}}{\rm{c}}{\rm{e}}\,{\rm{o}}{\rm{r}}\,{\rm{a}}{\rm{b}}{\rm{s}}{\rm{e}}{\rm{n}}{\rm{c}}{\rm{e}})=\frac{{\rm{ \% }}\,{\rm{o}}{\rm{f}}\,{\rm{s}}{\rm{i}}{\rm{n}}{\rm{k}}{\rm{h}}{\rm{o}}{\rm{l}}{\rm{e}}{\rm{s}}({\rm{p}}{\rm{r}}{\rm{e}}{\rm{s}}{\rm{e}}{\rm{n}}{\rm{t}}\,{\rm{o}}{\rm{r}}\,{\rm{a}}{\rm{b}}{\rm{s}}{\rm{e}}{\rm{n}}{\rm{t}})\,{\rm{i}}{\rm{n}}\,{\rm{a}}\,{\rm{s}}{\rm{u}}{\rm{s}}{\rm{c}}{\rm{e}}{\rm{p}}{\rm{t}}{\rm{i}}{\rm{b}}{\rm{i}}{\rm{l}}{\rm{i}}{\rm{t}}{\rm{y}}\,{\rm{c}}{\rm{l}}{\rm{a}}{\rm{s}}{\rm{s}}}{{\rm{ \% }}\,{\rm{o}}{\rm{f}}\,{\rm{t}}{\rm{o}}{\rm{t}}{\rm{a}}{\rm{l}}\,{\rm{a}}{\rm{r}}{\rm{e}}{\rm{a}}\,{\rm{c}}{\rm{o}}{\rm{v}}{\rm{e}}{\rm{r}}{\rm{e}}{\rm{d}}\,{\rm{b}}{\rm{y}}\,{\rm{t}}{\rm{h}}{\rm{e}}\,{\rm{s}}{\rm{u}}{\rm{s}}{\rm{c}}{\rm{e}}{\rm{p}}{\rm{t}}{\rm{i}}{\rm{b}}{\rm{i}}{\rm{l}}{\rm{i}}{\rm{t}}{\rm{y}}\,{\rm{c}}{\rm{l}}{\rm{a}}{\rm{s}}{\rm{s}}\,}$ was calculated for all susceptibility classes to compare the models.

## Results

### Sinkhole size distribution

**Figure 4:**
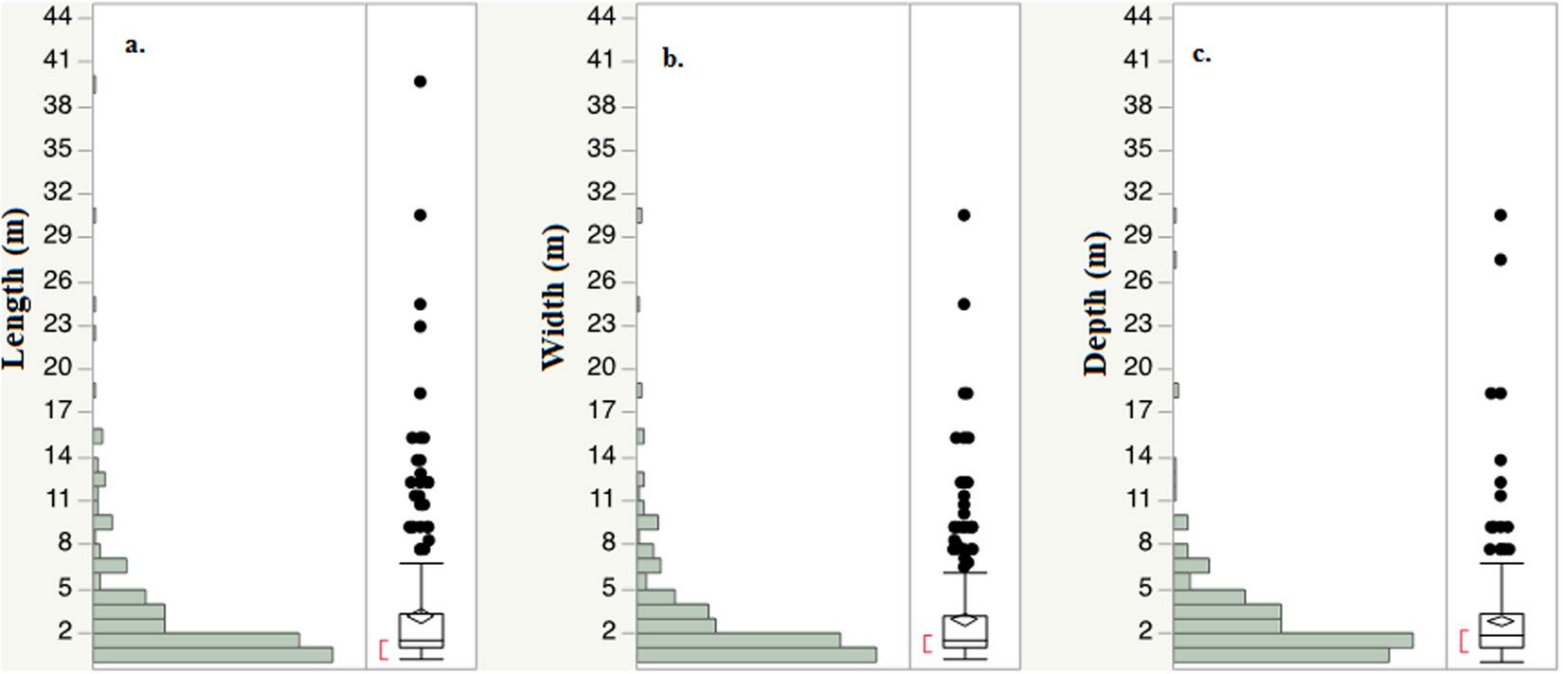
Sinkhole size distribution (**a**) length (m)- longest side of the feature (**b**) width (m)- shortest side of the feature, and (**c**) depth (m)- ground surface to the bottom of the feature. Only sinkholes with available dimensions in the sinkhole incident report are included.

Distribution of sinkhole dimension (length, width, or depth) had a log-linear distribution (Fig. 4). Of the total sinkholes with reported dimensions, about 55% of sinkholes were circular shaped. Elongated shaped sinkholes were about 14% and the rest (31%) were irregular shaped sinkholes. The maximum and the median diameter of circular sinkholes were respectively 24.8 m and 1.5 m. The largest elongated sinkhole reported had the maximum length of 39.6 m. The median length of the elongated sinkhole was 3 m for this study. Irregular shaped sinkholes had a maximum length of 30.5 m and a median length of 2 m. Sinkhole median depths for the circular, elongated, and irregular shaped were 1.8, 2.4, and 1.5 m respectively. Prevalence of circular sinkholes in the study area highlights that the karst topography is relatively young^68^.

### Model results

For AHP based susceptibility model, we provide the pairwise comparison matrix for the sub-criteria and data layers in Table 4. Normalization of principle eigen vector associated with the pairwise comparison matrix for predisposing factors resulted in the highest weight to surficial geology (0.426) followed by closeness to topographic depression (0.322), soil permeability (0.147), distance to flow channels (0.063), and distance to active mines (0.042). For the LR based model, parameter estimates of predisposing factors are provided in Table 5. The effect summary of the logistic regression model [LogWorth = −log_10_(pValue)] ranked the predisposing factors to sinkhole susceptibility as distance to closed topographic depression (8.021) > distance to active mines (3.9) > surficial geology (2.024) > distance to flowlines (1.493) > soil permeability (0.425). Variance inflation factor less than 10 suggested that there was no evidence of multi-collinearity among predisposing factors for this study. Confusion matrix for the LR model on the training data showed an overall accuracy of (73.07%) (Table 6). Though not significant in the LR model, we retained soil permeability data layer (p = 0.37) in the final model because a) its inclusion allowed reasonable comparison with AHP based approach used in this study, b) it was not correlated with any other factors, and c) its removal did not significantly improve the model performance for this study (area under the receiver operating characteristic (ROC) curve without soil permeability = 0.799 vs. 0.798 with soil permeability).

**Table 5:** Parameter estimates of the logistic regression based on the sinkhole presence/absence training data.

Parameters	Estimates	Variance inflation factor (VIF)
Intercept	−4.190611	—
Surficial geology		1.083
Undifferentiated TQ sediments	6.668699	
Beach, ridges, and dunes	−18.944975	
Holocene sediments	−18.497396	
Ocala LS	6.598917	
Cypresshead FM	5.354556	
Undifferentiated	6.500286	
Hawthorn FM	5.790065	
Coosawhatchie FM	6.609846	
Soil permeability	−0.005629*	1.353
Distance to closed topographic depression	−0.003223	1.171
Distance to active mines	−0.000095	1.289
Distance to flowlines	−0.000202	1.186

*Not significant (at 95% confidence level) in the model.

**Table 6:** Confusion matrix for the logistic regression on the training sinkhole presence/absence data.

Actual	Predicted		Correct percentage
	Presence	Absence	
Presence	101	67	60.12
Absence	59	241	80.33
Overall Accuracy			73.07

### Sinkhole susceptibility zonation

**Figure 5:**
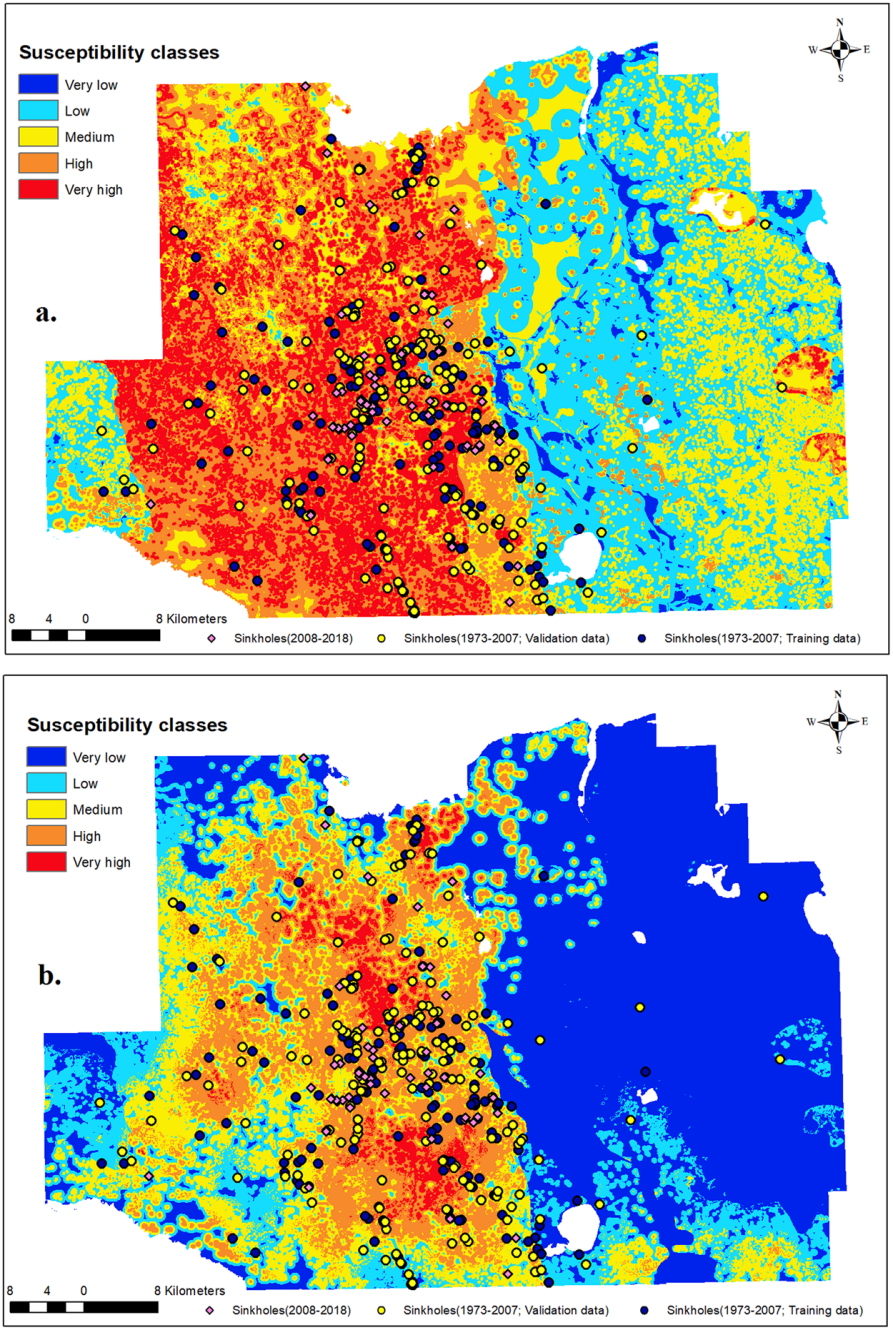
Sinkhole susceptibility maps for the study area based on a. the AHP based approach, and b. LR based approach.

Sinkhole susceptibility maps prepared using the AHP and LR based approaches are provided in Fig. 5. The AHP based approach resulted in susceptibility map with about 20.4% of the study area falling in very high susceptibility class. High susceptibility class covered almost 23% of the study area. Very low, low, and medium classes covered about 55.4% of the total area. In contrast, the LR based approach resulted in very high susceptibility class in about 2.9% of the total area. High susceptibility class only covered about 17.8% of the total area. Very low, low, and medium classes covered about 78% of the study area. When compared with the AHP based approach, there is a remarkable difference in area allocation to the very low and very high classes. While the AHP based approach tended to allocate more area to very high class and less area to very low class (2.7%), the LR based approach allocated more area to very low class (42%) and less area to very high class.

**Figure 6:**
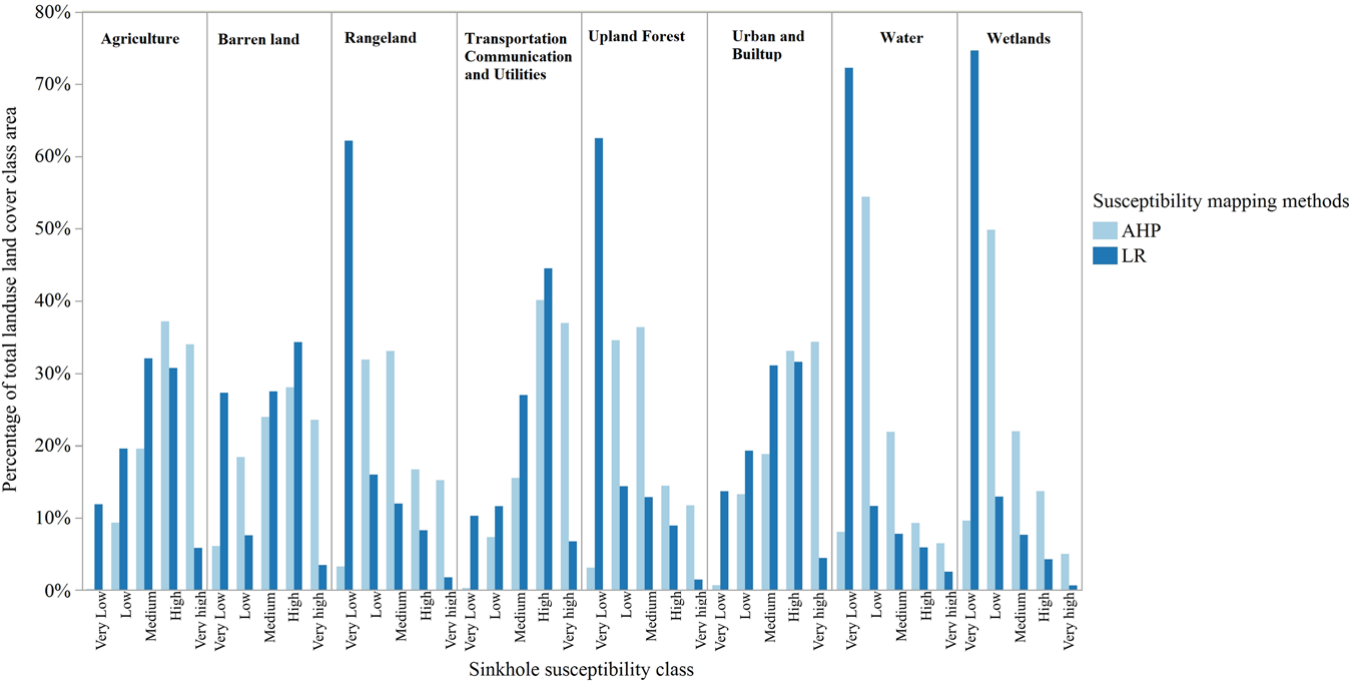
Percentage of total land use land cover class area falling in each sinkhole susceptibility class mapped by AHP and LR based approaches. For each land use land cover class (e.g. agriculture), the bars represent % total land use land cover area of the same class (agriculture) represented by each sinkhole susceptibility class (very low, low, medium, high, and very high).

We assessed the vulnerability of urban residential areas and other land use land cover classes of the study area to sinkhole susceptibility by extracting susceptibility classes using the masks of land use land cover classes. Land use land cover classes were obtained from the Florida Department of Environmental Protection (2017). For the study area, these landuse land cover data were compiled by Florida Department of Environmental Protection from digital orthophotographs taken in 2013–2014. ‘Statewise_Land_Use_Land_Cover.shp’ vector data file available online at: http://publicfiles.dep.state.fl.us/otis/gis/data/STATEWIDE_LANDUSE.zip was used in this study. We converted major land use class (Level I- classification representing general land use land covers; Florida Department of Transportation, 1999^69^) polygons in the statewise land use land cover shapefile to raster with the pixel resolution of 30 m × 30 m in ArcGIS 10.3.1 software (Environmental Systems Research Institute, Inc., Redlands, CA, 2015). The AHP based mapping resulted in almost 67% of urban and built-up land cover class falling in high and very high susceptibility zones compared to about 36% when the LR based approach was used (Fig. 6). Likewise, for the agriculture land cover, AHP based approach resulted in almost 71% of the area being under high and very high classes compared to about 37% from the LR based approach. Among land cover classes, transport and utilities class had the highest percentage of its total area under high and very high susceptibility class for both approaches (AHP = 75% vs. LR = 47%).

### Model evaluation and comparison

**Figure 7:**
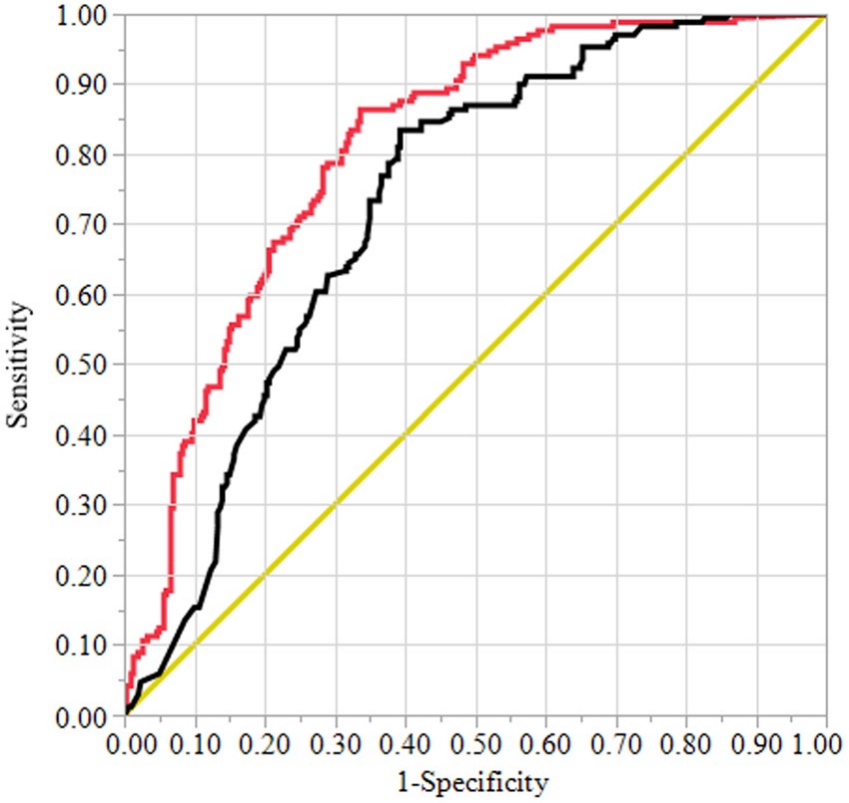
ROC curves for the AHP based (black color) and LR based (red color) models for sinkhole susceptibility using the validation data (1973–2007). Diagonal represents 1:1 line between sensitivity and 1-specificity. Area under the curve was 0.730 for the AHP based model and 0.808 for the LR based model.

**Figure 8:**
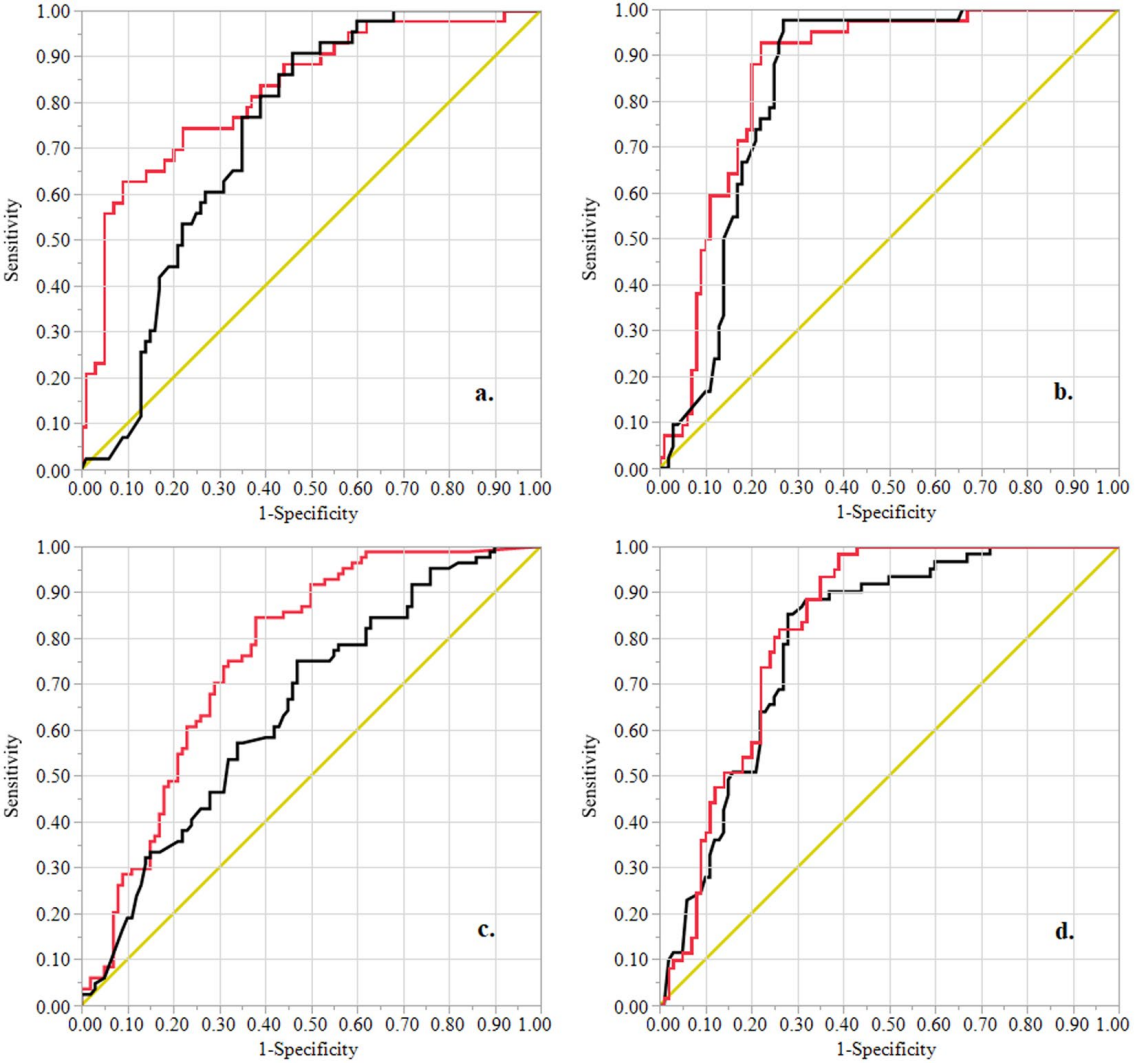
ROC curves for the AHP based (black color) and LR based (red color) model to sinkhole susceptibility using temporal validation data- (**a**). 1973–1986 (AUC: AHP = 0.734, LR = 0.826), (**b**). 1987–1998 (AUC: AHP = 0.831, LR = 0.860), (**c**). 1999–2007 (AUC: AHP = 0.649, LR = 0.760), (**d**). 2008–2018 (AUC: AHP = 0.796, LR = 0.826). Diagonal represents 1:1 line between sensitivity and 1-specificity. Note that the LR model training data only included sinkholes between 1973 and 2007.

We evaluated both models using the area under the ROC curve approach. On the validation data set (1973–2007), the area under the ROC curve for the AHP based approach was almost 9.6% smaller than the area under the ROC curve for the LR based approach (Fig. 7). The LR based approach was significantly better than the AHP based approach (Table 7). Both modeling approaches performed well in identifying sinkhole susceptibility zones for the study area on evaluated time periods (Fig. 8). Between the three time periods evaluated, the LR based approach performed better than the AHP based approach. While AHP based approach could successfully predict about 73%, 83%, and 65% of the presence and absence of sinkholes in the validation data, the LR based approach explained about 83%, 83%, and 76% respectively for 1973–1986, 1987–1998, and 1999–2007 (Fig. 8a–c). Both models predicted future sinkhole incidents in our study area relatively well. For instance, the area under the ROC curve for the AHP and LR based approaches were about 0.8 and 0.83 respectively for the period of 2008–2018 (Fig. 8d).

**Table 7:** Area under the ROC curve for the AHP vs. LR based sinkhole susceptibility model and their comparison.

Model	AUC	Std. Error	Lower 95%	Upper 95%	χ^2^	Prob > χ^2^
AHP	0.7297	0.0230	0.6823	0.7723	—	—
LR	0.8083	0.0199	0.7663	0.8443	—	—
**Test of AUC difference**						
LR-AHP	0.0786	0.0181	0.0431	0.1142	18.84	<0.0001

**Figure 9:**
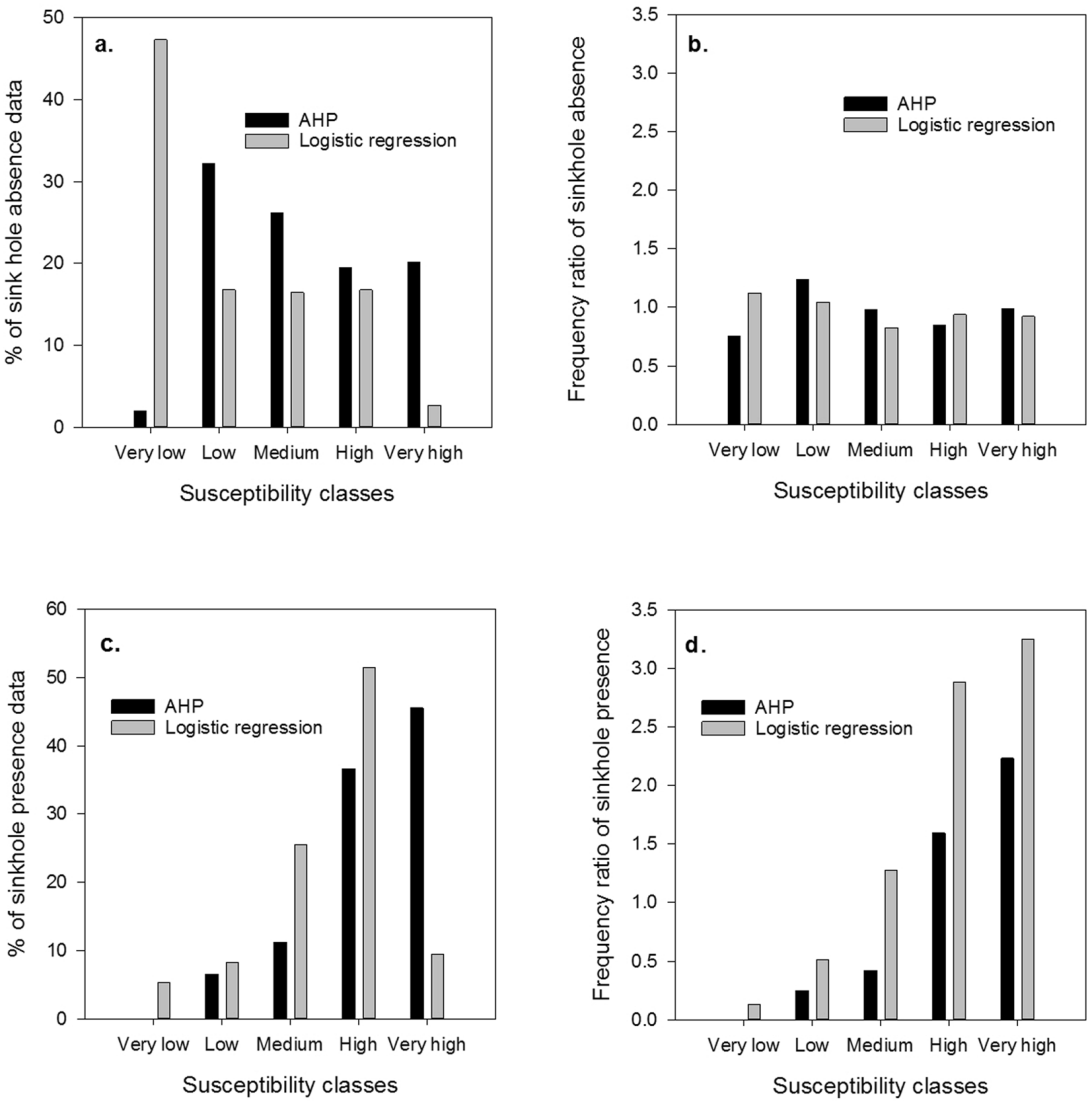
Evaluation of susceptibility classes (equal area classification) for AHP based and LR based maps to identify sinkhole absence (**a,b**) and presence (**c,d**) on the validation data. Frequency ratio of presence (or absence) for a susceptibility class was estimated as the ratio of % of total sinkhole presence (or absence) to % of total area represented by that class.

We also evaluated the susceptibility classes for the AHP and LR based maps to positively identify sinkhole absence and presence in the study area using the validation data. When compared with the sinkhole absence data, the very low susceptibility class of the AHP was inferior to LR based approach in identifying sinkhole absence (Fig. 9a,b). For example, both the percentage of sinkhole absence data and frequency ratio for the very low susceptibility class was lower than in other classes. However, the LR based approach was better able to address this problem observed for the AHP based approach. For instance, both the percentage of sinkhole absence and frequency ratio were higher for the very low susceptibility classes than other classes. However, the AHP based and LR based susceptibility classes performed well on the sinkhole presence data (Fig. 9c,d). For example, the percentage of sinkholes falling on each class increased with its susceptibility to sinkhole occurrence for the AHP based approach. For the LR based approach, the percentage of sinkholes was highest in the high susceptibility class. Nevertheless, highest frequency ratio for the sinkhole presence in the very high susceptibility class in the LR based susceptibility map suggests that this class could discriminate areas of high and very high sinkhole occurrence relatively well.

## Discussion

Identifying areas sensitive to sinkhole formation is important typically in Florida because of its hydro-geologic setting and karst topography. The strong dependence of Florida on groundwater for its water needs makes it critical to identify sinkhole hazard zones and minimize the effects of anthropogenic processes on sinkhole formation and groundwater contamination^5,6^. In that context, this study compares the applicability of two common approaches to sinkhole susceptibility mapping by utilizing publicly available long-term subsidence incident record for Marion County located in central Florida and discusses potential limitations to these approaches. This study also evaluates the predictive capability of the models (generated using past sinkhole incidents) to successfully map potential areas of future sinkholes by using independent sinkhole incidents occurring at later dates.

Both the AHP and LR models of sinkhole susceptibility mapping suggested that urban areas, agricultural areas, and transport utilities had higher potential for sinkhole activity in our study area. The distribution of existing sinkholes is primarily concentrated in the Ocala area and along major highways. The U.S. Census Bureau estimated that almost 59,110 people lived in 26,081 housing units with the median housing value of $120,700 in Ocala city in 2017. Therefore, a significant risk of sinkhole associated loss of property and lives exists for this part of the study area. Spatial location of sinkhole susceptible zones in these areas are likely due to the pressure of urban water consumption on the aquifer or the presence of carbonate and dolomitic geology (Ocala formation)^48,68^. It is likely that further development and urbanization will affect sinkhole distribution in this area^68^. However, there were inherent differences in the area of land cover groups being classified as being very vulnerable to sinkhole susceptibility under the AHP and LR based methods. Differences in the relative contribution of predisposing factors to sinkhole susceptibility between two mapping methods (subjective judgment in AHP vs. training datasets in the LR model) likely resulted in observed differences in susceptibility in our study.

Surficial geology was the predominant contributor to sinkhole formation (43% contribution) compared to other predisposing factors for the AHP model. Other qualitative susceptibility mapping studies in various geographic regions also placed more weight on underlying geology for sinkhole formation^17,70,71^. For example, Todd and Ivey-Burden^71^ allocated almost 60% relative weight to bedrock compared to other predisposing factors when mapping sinkhole susceptibility in Virginia. Likewise, Taheri *et al*.^17^ also assigned most weight to bedrock lithology (34%) compared to other predisposing factors like distance to faults, groundwater withdrawal, distance to deep wells, the thickness of alluvium etc. in Iran. In the LR model, however, closeness to topographic depressions rather than surficial geology was the most important variable. It is likely that closer to topographic depressions, underlying geo-physical factors form a conducive environment to sinkhole formation. Prior studies have also suggested that, in karst topography, the formation of a sinkhole favors the occurrence of additional subsidence events due to changes in subsurface conditions^20,54,72,73^. Zhou *et al*.^20^, for example, found that sinkholes were likely to occur within a 30 m radius of existing sinkholes in areas underlain by carbonate rocks in Maryland.

The AHP based model explained the presence of sinkhole incidents reasonably well in our study. For example, the AHP based model predicted almost 43% of sinkhole occurrence in the validation data within 20% of the highest susceptibility value for this study. A similar degree of prediction (32–48%) was obtained by Galve *et al*.^23^ when using a heuristic model for cover-collapse sinkholes in evaporite karsts. The AHP based approach, however, required multiple evaluations through trial-and-error process to generate logically consistent relative weights to better predict existing sinkhole incidents^17^. One of the caveats of this trial and error approach, however, is the introduction of bias towards sinkhole presence. Consequently, this approach may result in larger areas in higher sinkhole susceptibility classes often limiting its applicability in sinkhole risk mitigation strategies^17^. In our study, almost 43% of the total study area fell under high to very high susceptibility class, which may potentially limit its applicability in hazard risk mitigation responses to a regional scale.

Qualitative and semi-qualitative approaches to sinkhole susceptibility mapping depend on correctly identifying predisposing factors and their relative contributions to sinkhole formation^17,27,32^. One of the major challenges to AHP based approach is uncertainty in prioritizing predisposing factors to sinkhole formation. For instance, ranking relative contributions becomes difficult when the factors are many and little is known about the spatial contribution of these factors to sinkhole occurrence. However, calculation of the frequency distribution of the existing sinkholes in different sub-criteria or generation of the cumulative distribution function could aid in the determination of relative importance of sub-criteria on sinkhole formation^70^. Using these methods to guide pairwise comparisons may not necessarily reflect the importance of that criterion to sinkhole formation, but just a representation of the spatial distribution of sinkholes in each criterion. Since complex interactions among several factors contribute to the formation of sinkholes, an overly simplified model such as the AHP model of ours serves in rapid assessment of sinkhole susceptibility at a regional scale.

Logistic regression approaches to susceptibility mapping have been widely used across different hazard-prone regions to reliably predict natural hazards like landslides^27,29,30,74^ and sinkholes^8,24^. The LR based model generated in this study performed well in mapping sinkhole susceptibility for the study area. Within 20% near the highest susceptibility value, our model predicted 56.8% of the total sinkholes in the validation data. Similar to our study, in an evaporite karst in northeast Spain, Galve *et al*.^23^ predicted about 59% of total sinkholes within 20% of the highest susceptibility by using probabilistic models for cover-collapse sinkhole. Within 40% near the highest susceptibility, almost 85.2% of sinkholes were predicted by the LR based model in our study. In addition, the area under the ROC curve value > 0.8 suggested a goodness of fit for sinkhole susceptibility mapping for this study. While there are relatively little LR based sinkhole susceptibility mapping studies for this region, the study by Kim and Nam^75^ for central Florida do not report validation of their LR model. Nevertheless, our LR model performed similar to the models reported by Ciotoli *et al*.^24^ for sinkhole susceptibility for Lazio Region in central Italy (AUC = 0.779) and by Ozdemir^76^ for Karapinar region in Turkey (AUC = 0.814).

In probabilistic susceptibility models, previous studies have demonstrated that accounting for clustering of sinkholes improved model performance^16,23^. We observed a significant clustering of sinkholes with the nearest neighbor ratio of 0.63 in our study (p < 0.001). Incorporating variables, such as nearest sinkhole distance, into the probabilistic model has been shown to improve model performance in other regions^16^. A previous study by Shofner *et al*.^54^ suggested that topographic depressions could serve as an index of sinkhole clustering or surface karstification on a regional scale. We expect only a little improvement with the addition of clustering variable in our LR model because inclusion of ‘the distance to closed topographic depressions’ as one of the variables in our study potentially accounted for clustering effect. Results from the LR modeling also support this interpretation because the closeness to topographic depression, not surficial geology, was the most important variable to explain sinkhole presence or absence in our study.

## Limitations and Future Work

The scale of the study, data unavailability for the whole study area (e.g., the thickness of overburden, aquifer recharge), and associated costs to collect these data restricted predisposing factors in our model to relatively few. Future works should, therefore, emphasize the inclusion of other predisposing factors like groundwater withdrawal^47^, depth to the water table^9,48^, thickness of overburden^20^, and recharge of aquifers^50^ to develop robust models for this region. Since human activities influence factors like groundwater withdrawal, depth to water table, and recharge of the aquifer, including these factors in the sinkhole modeling approaches would take into account variabilities introduced by urban sprawl in sinkhole occurrence and distribution^68^.

The use of absence of hazard often creates challenges to using LR method in susceptibility mapping of natural hazards. Most studies assume that areas without true hazard to be the true absence of a hazard. However, this does not necessarily mean hazard may not be possible in the future. For our study, randomly generated sinkhole absence data points were evaluated to be ‘true absence’ using three criteria: (a) locations were different from the points reported as sinkhole incidents, (b) multiple time series evaluation of the aerial image and high resolution google earth image (1999–2018) showed no remarkable features indicative of sinkholes (e.g., circular/elongated depressions), and (c) these points were not located in closed depressions (using 1.5 m contours) in areas covered by dense forest (e.g., eastern part of the study area). As aerial images/satellite images used were from 1999 onwards, our approach of identifying sink hole absence may introduce some errors if sinkhole occurred in ‘true absence’ locations before 1999. We suggest that future work on LR modeling of sinkholes should also focus on evaluating truly absent areas of sinkhole occurrence through the use of modern techniques like LiDAR or RADAR^77–79^.

Identifying and maintaining long-term sinkhole inventory is critical to modeling and validating sinkhole susceptibility models. This study relied on the publicly available subsidence incident report maintained by the FDEP-FGS. One of the advantages of using the subsidence incident report was a comprehensive record of user reported sinkhole incidents, including location, date, time, shape, size, dimensions, predisposing factors, land use, closeness to existing sinkholes, etc. However, some of these subsidence incidents may not have been verified as ‘true sinkholes’ by geologists (FDEP-FGS, 2011). Based on the incident report, the long-term average (1973–2018) sinkhole occurrence rate of about 9 per year was estimated for our study area. We expect this number to be much higher because (a) most of the eastern region of the study area is covered by Ocala national forest and sinkholes in this region are less likely to be observed and reported by the users, (b) some users may be reluctant to report sinkhole formation on their private property due to the negative effect on real estate values, and (c) sinkhole incident reporting is not mandatory in Florida. Though the validity of these data against reporting bias has already been examined by Fleury *et al*.^80^ and this database has successfully been used by other studies^68,81^, future works should focus on updating sinkhole database for the eastern region of the study area to eliminate potential bias. Use of LiDAR assisted sinkhole detection methods, which have shown a strong promise in detecting sinkholes in inaccessible areas including under the forest canopy cover^79^, could supplement the use of existing sinkhole incident reports in susceptibility mapping.

## Conclusion

We presented the applicability of two common sinkhole susceptibility mapping approaches for an area with prominent karst topography in central Florida. Of the two mapping approaches used in this study, the LR based approach was superior to the AHP based approach in successfully identifying potential sinkhole zones. Nevertheless, the performance of the AHP based approach was reasonable for this study considering its applicability in rapid and regional assessment. The LR based approach used in this study suggested that closeness to existing topographical depression is the most important predisposing factor for sinkhole susceptibility. Surficial geology and distance to flow networks were also important predisposing factors for sinkhole susceptibility for this study. Sinkhole susceptibility mapping revealed that the majority of the urban residential areas (35–64%) in the study area fell under high to very high sinkhole risk zones. This signifies that proper mitigation approaches and hazard response mechanisms may be necessary to minimize the risks associated with sinkhole formations in these areas. In that context, the prospects of integration of probabilistic and heuristic approaches with GIS in sinkhole susceptibility zonation are good. While the mapping approaches described in this study are applicable in other areas, site-specific validation of these models should be made prior to application.

## Supplementary information

Supplementary Information

## Data Availability

The datasets used in the current study are publicly available from Florida Department of Environmental Protection, Florida Geological Survey, USDA, and USGS. Relevant datasets are also available from the corresponding author on reasonable request.
