# Supplementary material for: Sinkhole susceptibility mapping in Marion County, Florida: Evaluation and comparison between analytical hierarchy process and logistic regression based approaches
Source: Sci Rep. 2019 May 9;9:7140. doi: 10.1038/s41598-019-43705-6 (PMC6509126; doi:10.1038/s41598-019-43705-6)
Supplement: Supplementary file 1 — Supplementary Information [file 41598_2019_43705_MOESM1_ESM.docx]

**Supplementary information for article**

**Sinkhole susceptibility mapping in Marion County, Florida: Evaluation and comparison between analytical hierarchy process and logistic regression based approaches**

Praveen Subedi^1^, Kabiraj Subedi^2*^, Bina Thapa^3^, Pradeep Subedi^4^

^1^School of Forest Resources and Conservation, University of Florida, Gainesville, Florida; ^2^Department of Geography, Prithivi Narayan Campus, Tribhuvan University, Pokhara, Nepal; ^3^Department of Natural Resource Ecology and Management, Iowa State University, Ames, Iowa; ^4^Rutgers Discovery Informatics Institute, Rutgers University, Piscataway, New Jersey.

*corresponding author ([kabisubedi1275@gmail.com](mailto:kabisubedi1275@gmail.com))


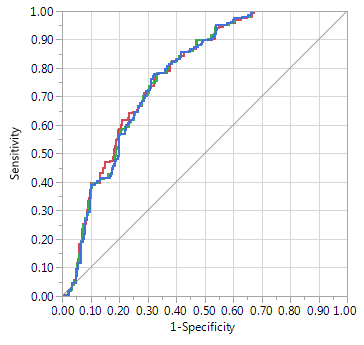


**Supplementary Figure S1.** Comparison of receiver operating characteristic (ROC) curves among unbalanced (presence:absence = 1: 1.78) logistic regression model, balanced (under-sampled) logistic regression (average model of 10 balanced datasets), and weighted (higher weights assigned to presence data) logistic regression model for sinkhole occurrence. Red line shows the LR from unbalanced data, blue line shows LR averaged from 10 balanced (under-sampled) data, and green line shows weighted LR model to balance the presence data. The area under the ROC curve (AUC) for the unbalanced LR model is 0.781, AUC for the balanced LR model is 0.776, and the weighted LR model is 0.777.
